# Association of Continuous Glucose Monitoring with Glycaemic, Distress, and Quality of Life Outcomes in Insulin-Treated Type 2 Diabetes: A 12-Month Prospective Study

**DOI:** 10.3390/medicina62050938

**Published:** 2026-05-11

**Authors:** Klara Dzojic, Ema Schönberger, Matea Petrinović, Daria Sladić Rimac, Ema Erzic, Maja Cigrovski Berkovic, Miro Bakula, Ines Bilić-Ćurčić, Silvija Canecki-Varžić

**Affiliations:** 1 Department of Endocrinology, Clinical Hospital Centre Osijek, 31000 Osijek, Croatia; kormanac@mefos.hr (K.D.); ema.schonberger7@gmail.com (E.S.); mateapetrinovic@gmail.com (M.P.); daria.rimac@gmail.com (D.S.R.); silvija.canecki@gmail.com (S.C.-V.); 2 Faculty of Medicine, Josip Juraj Strossmayer University of Osijek, 31000 Osijek, Croatia; 3 Department of Nursing and Palliative Medicine, Faculty of Dental Medicine and Health Osijek, Josip Juraj Strossmayer University of Osijek, 31000 Osijek, Croatia; 4 Faculty of Kinesiology, University of Zagreb, 10000 Zagreb, Croatia; maja.cigrovskiberkovic@gmail.com; 5 Department of Internal Medicine, Division of Endocrinology, Diabetes and Metabolic Diseases, Sveti Duh University Hospital, 10000 Zagreb, Croatia; mbakula@kbsd.hr

**Keywords:** type 2 diabetes, CGM, insulin therapy, HbA1c, distress, quality of life

## Abstract

*Background and Objectives*: Continuous glucose monitoring (CGM) improves glycemic control in type 2 diabetes (T2DM), but its effects on diabetes-related distress and quality of life (QoL), particularly in patients on intensive insulin therapy, are less well studied. Aim: We aim to assess the impact of CGM on glycemic control, diabetes distress, and QoL in adults with T2DM on intensified insulin therapy. *Materials and Methods*: This prospective observational study included 226 adults with T2DM using multiple daily insulin injections or basal–bolus therapy. CGM was initiated at baseline. HbA1c, fasting glucose, and lipid profile were measured at baseline, 3, 6, 9, and 12 months. Diabetes-related distress (DDS-17) and quality of life (MDQoL-17) were assessed at the same time points. Longitudinal changes were analyzed using linear mixed-effects models. *Results*: Mean age was 66 ± 9.1 years; 55% were male. HbA1c decreased from 8.56 ± 1.87% to 7.20 ± 0.90% at 3 months (*p* < 0.001) and remained improved at 12 months (7.21 ± 1.04%). Diabetes distress declined significantly over time (β = −0.025/month; *p* = 0.001). Older age and lower income were associated with higher distress. Quality of life improved significantly during follow-up; higher income predicted better QoL, while greater distress predicted poorer QoL. HbA1c did not independently influence QoL. CGM metrics (GMI, mean glucose, TIR, glycemic variability) remained stable after initial improvement. *Conclusions*: In 226 insulin-treated T2DM patients, implementation of CGM as part of a structured insulin intensification strategy was associated with sustained improvements in glycaemic control, reduced diabetes-related distress, and enhanced quality of life over 12 months. These findings support routine CGM use and highlight the importance of addressing psychosocial outcomes in diabetes care.

## 1. Introduction

Diabetes mellitus type 2 (T2DM) remains a major global health challenge, characterized by progressive β-cell dysfunction, increasing insulin requirements, and mounting risk of micro- and macrovascular complications. As the disease advances, many patients eventually require intensified insulin therapy—including multiple daily injections (MDIs) or basal–bolus regimens—to achieve and maintain glycaemic targets. However, despite treatment escalation, glycaemic control frequently remains suboptimal, and the burden of insulin therapy (e.g., hypoglycaemia risk, regimen complexity) frequently contributes to psychological distress and impaired quality of life (QoL) [1,2].

In recent years, continuous glucose monitoring (CGM) technologies have emerged as a promising tool to enhance glycaemic management by providing real-time feedback on glucose levels, trends, and variability. Most evidence to date has focused on people with type 1 diabetes or those using insulin pump therapy, while the use of CGM in T2DM patients treated with MDI has been relatively understudied. A landmark randomized clinical trial in adults with T2DM receiving MDI found that CGM use resulted in a modest but statistically significant reduction in HbA1c (−0.3% at 24 weeks), albeit without meaningful differences in hypoglycaemia or QoL outcomes [3,4]. More recently, a randomized study in T2DM patients treated with basal insulin (without prandial injections) demonstrated that CGM versus self-monitoring of blood glucose (SMBG) led to a significant HbA1c reduction at 8 months [5]. A systematic review published in 2024 concluded that CGM may lead to better clinical outcomes than SMBG in T2DM but highlighted that evidence on patient-reported outcomes (PROMs) remains inconclusive [6].

Beyond glycaemic metrics, the psychosocial dimension of diabetes management—particularly diabetes-related distress and health-related quality of life (QoL)—has increasingly been recognized as a critical component of comprehensive care [1,2]. Insulin therapy, while essential for achieving metabolic control, may impose additional burdens, including regimen complexity, fear of hypoglycaemia, and reduced flexibility in daily life, all of which can adversely affect QoL [7,8]. Although some studies suggest that improvements in glycaemic control may indirectly enhance QoL in insulin-treated patients with type 2 diabetes, the relationship remains inconsistent and appears to be mediated by psychosocial rather than purely metabolic factors [9,10]. Importantly, the direct effects of glucose monitoring technologies, including continuous glucose monitoring, on psychological outcomes in this population remain insufficiently characterized, with limited and heterogeneous existing evidence [11,12].

In addition to glycaemic metrics, contemporary diabetes care increasingly emphasizes patient-centered outcomes, including time in range (TIR), treatment burden, and psychosocial well-being [13,14]. Emerging frameworks position continuous glucose monitoring (CGM) not only as a monitoring tool but also as a behavioral and digital therapeutic intervention that may influence self-management, emotional burden, and perceived disease control [8,12,15]. Furthermore, CGM-derived metrics such as TIR and glycaemic variability are now recognized as clinically meaningful endpoints associated with improved outcomes and are increasingly incorporated into international consensus recommendations [13,14]. However, longitudinal real-world data integrating both metabolic and psychosocial outcomes in insulin-treated T2DM populations remain limited, with existing evidence largely derived from short-term randomized studies or heterogeneous observational cohorts [5,6].

Therefore, the aim of this study was to evaluate the impact of CGM implementation as part of an intensified insulin therapy strategy in adults with T2DM, with a dual focus on glycaemic outcomes and patient-reported measures, including diabetes-related distress and quality of life. By combining longitudinal clinical and psychosocial data, this study seeks to provide a more comprehensive understanding of the role of CGM in contemporary diabetes care.

## 2. Materials and Methods

### 2.1. Study Design

This was a prospective, observational longitudinal study conducted at a tertiary diabetes center. In total, 226 participants were followed for 12 months, with clinical and psychosocial assessments performed at baseline (0 months), and at 3, 6, 9, and 12 months.

At baseline, all participants were introduced to a continuous glucose monitoring (CGM) system as part of intensification of insulin therapy. Routine diabetes education and insulin dose adjustments were provided according to clinical judgment. CGM was introduced as part of a broader therapeutic intensification strategy, which included insulin dose optimization and structured patient education. CGM data were obtained using FreeStyle Libre/FreeStyle Libre 2 systems (Abbott Diabetes Care, Witney, UK) and Dexcom ONE systems (Dexcom Inc., San Diego, CA, USA). CGM metrics were analyzed using manufacturer-supported software platforms (LibreView https://www.libreview.com/, Abbott Diabetes Care, Witney, UK; Dexcom Clarity https://clarity.dexcom.eu/, Dexcom Inc., San Diego, CA, USA). 

### 2.2. Participants

#### 2.2.1. Inclusion Criteria

- Adults (≥18 years) with type 2 diabetes mellitus,
- On intensified insulin therapy (multiple daily injections or basal–bolus regimen),
- Diabetes duration ≥ 1 year,
- Willingness to wear CGM and complete follow-up visits,
- Ability to understand and provide informed consent.

#### 2.2.2. Exclusion Criteria

- Type 1 diabetes or other specific types of diabetes,
- Severe cognitive impairment, psychiatric disorders, or inability to complete questionnaires,
- Pregnancy or lactation,
- Incomplete baseline data.

### 2.3. Intervention: Continuous Glucose Monitoring (CGM)

At baseline, participants received professional or real-time CGM systems. Patients were instructed in sensor insertion, scanning procedures, calibration (if applicable), and interpretation of glucose trends. Insulin doses were adjusted according to sensor-derived glucose profiles and routine clinical assessments.

CGM-derived variables included: glucose management indicator (GMI), time in range (TIR; 3.9–10.0 mmol/L), time above range (TAR), time below range (TBR) and coefficient of variation (CV [%]).

### 2.4. Clinical Data Collection

At each study visit (0, 3, 6, 9, 12 months), venous blood samples were taken for the measurement of HbA1c, fasting plasma glucose, body weight and BMI, lipid profile (total cholesterol, LDL, HDL, triglycerides), serum creatinine and estimated GFR, and albumin-to-creatinine ratio (UACR). All samples were analyzed using standardized laboratory techniques.

Baseline demographic variables included age, sex, duration of diabetes, comorbidities, income level, education, smoking and alcohol consumption habits. Socioeconomic variables included self-reported monthly income category and education level. These variables were included to explore contextual determinants of diabetes-related distress and quality of life.

### 2.5. Psychosocial Measures

#### 2.5.1. Diabetes Distress

Diabetes-related distress was assessed using the Diabetes Distress Scale (DDS-17), a validated 17-item self-report measure of diabetes-specific emotional distress [8]. The instrument assesses four domains: emotional burden, physician-related distress, regimen-related distress, and interpersonal distress. Each item is scored on a 6-point Likert scale ranging from 1 (“not a problem”) to 6 (“a very serious problem”). The overall DDS score was calculated as the mean of all 17 item responses, with higher scores indicating greater diabetes-related distress. The DDS-17 was administered at baseline and at 3, 6, 9, and 12 months during follow-up.

#### 2.5.2. Quality of Life

Quality of life specific to diabetes was measured using the MDQoL-17 questionnaire, which contains 17 items across seven domains (Physical functioning, Emotional well-being, Role limitations, Energy/Fatigue, Social functioning, and General health) [16]. Scores were converted into percentage values (0–100%), where higher scores indicate better quality of life. QoL was evaluated at the same intervals as DDS.

### 2.6. Ethical Considerations

The study was conducted in accordance with the Declaration of Helsinki and approved by the Ethics Committee of Clinical Hospital Centre Osijek, Croatia, protocol code R1-817/2024, approval date 25 January 2024. Written informed consent was obtained from all participants before inclusion in the study.

### 2.7. Statistical Analysis

Descriptive statistics were used to summarize baseline clinical and demographic data. Longitudinal changes in metabolic, laboratory, and CGM-derived variables were summarized using medians and interquartile ranges. Pairwise comparisons between baseline and follow-up visits, or between available CGM follow-up time points, were performed using the Wilcoxon signed-rank test. Linear mixed-effects models with a random intercept for participants were used as the primary inferential approach for longitudinal outcomes, as this method accounts for within-subject correlation and allows inclusion of participants with incomplete follow-up data under the missing-at-random assumption. Time was included as a fixed effect. For glycaemic outcomes, CGM-derived metrics were additionally included as fixed effects. For diabetes-related distress and quality of life, relevant demographic and socioeconomic variables were included as covariates. Model estimates are presented as regression coefficients (β) with corresponding *p*-values. Statistical significance was set at *p* < 0.05. All analyses were conducted using IBM SPSS Statistics, version 25.0 (IBM Corp., Armonk, NY, USA).

## 3. Results

The study included 226 participants 90 (39.8%) females and 136 (60.2%) males aged 37–87 years, with longitudinal follow-up over 12 months. The study population represents a clinically complex group of insulin-treated patients with longstanding type 2 diabetes mellitus (T2DM). The baseline characteristics of the group are presented in Table 1.

Following initiation of CGM-supported care, participants demonstrated significant improvements in several metabolic parameters over the 12-month follow-up period (Table 2).

The most pronounced and consistent effect was observed for glycaemic control. HbA1c decreased significantly from baseline (median 8.1%) to 3 months (7.2%; *p* < 0.001), with sustained improvement maintained at 6, 9, and 12 months (all *p* < 0.001 vs. baseline). Fasting plasma glucose also improved early after CGM initiation (*p* = 0.010 at 3 months), although this effect was not consistently maintained at later time points.

In addition to glycaemic outcomes, significant reductions were observed in lipid parameters. Total cholesterol and triglycerides decreased significantly at all follow-up time points compared with baseline (all *p* ≤ 0.003), suggesting a favorable cardiometabolic profile associated with treatment intensification. In contrast, HDL cholesterol remained stable throughout follow-up, while LDL cholesterol showed a small transient increase at 3 months (*p* = 0.021) without sustained significance thereafter.

Anthropometric parameters showed modest and inconsistent changes. Body weight increased slightly at 3 and 6 months (*p* = 0.043 and *p* = 0.001, respectively), followed by a gradual reduction by 12 months; however, these changes were not statistically significant at later time points. Similarly, BMI changes were small and did not demonstrate a consistent or clinically meaningful trend.

Renal parameters remained stable over time. Estimated glomerular filtration rate (eGFR) did not change significantly across follow-up, and although urinary albumin-to-creatinine ratio (UACR) decreased significantly at 3 months (*p* = 0.047), this effect was not sustained at subsequent time points.

CGM-derived glucose metrics remained largely stable between 3 and 12 months, indicating maintenance of glycaemic control following the initial improvement observed after CGM initiation (Table 3).

Glucose management indicator (GMI) did not change significantly over time, with median values remaining consistently around 7.0–7.1% across all follow-up points (all *p* > 0.3). Similarly, time in range (TIR) remained stable throughout follow-up, with median values between 71.0% and 73.5%, and no statistically significant differences compared with 3 months.

Measures of glycaemic variability also showed no meaningful changes. The coefficient of variation (CV) remained stable across all time points (all *p* > 0.9), indicating consistent glucose variability after the initial treatment adaptation phase.

Time above range (TAR) and time below range (TBR) demonstrated small but statistically significant changes at 12 months compared with 3 months (*p* = 0.006 and *p* = 0.017, respectively). However, the absolute differences were modest and are unlikely to be clinically meaningful.

In Table 4, results were obtained using linear mixed-effects models with a random intercept for participants, examining outcomes (HbA1c, distress, and quality of life) in relation to predictors including time, GMI, TAR, CV, age, income, and distress.

In linear mixed-effects models, HbA1c showed a downward trend over time (β = −0.016 per visit, *p* = 0.076). Although this did not reach conventional statistical significance, the direction and consistency of change indicate an overall improvement in glycaemic control during follow-up.

Among CGM-derived metrics, GMI was the strongest predictor of HbA1c (β = +0.602, *p* < 0.001). Higher time above range (TAR) and greater glycaemic variability (CV) were also associated with higher HbA1c, although these effects were smaller. Time in range (TIR) was not independently associated with HbA1c.

Psychosocial variables, including diabetes distress and quality of life, were not significantly associated with HbA1c in the adjusted models. Diabetes-related distress significantly decreased over time (β = −0.025 per month, *p* = 0.001), indicating a progressive reduction in emotional burden during follow-up. Higher age was associated with greater distress (β = +0.022, *p* = 0.002), while higher income was associated with lower distress (β = −0.666, *p* = 0.027). Other clinical and demographic variables were not significantly associated with distress.

Quality of life improved significantly over time, with a clear longitudinal effect of follow-up duration. Among predictors, higher diabetes-related distress was strongly associated with poorer quality of life, while higher income was associated with better quality of life. Other socio-demographic and clinical variables did not show significant independent associations.

## 4. Discussion

The present study provides longitudinal real-world evidence that CGM-supported care in insulin-treated type 2 diabetes is associated with improvements not only in glycaemic control but also in diabetes-related distress and quality of life. Notably, the dissociation between HbA1c and quality of life observed in our cohort highlights the importance of psychosocial determinants in diabetes management, supporting a shift toward more patient-centered outcome frameworks.

We observed a significant and sustained reduction in HbA1c, with the greatest improvement occurring within the first 3 months following CGM initiation and subsequent stabilization over time. This pattern suggests that the greatest glycaemic benefit occurred early after CGM initiation and was subsequently maintained over time. This pattern is consistent with previous randomized trials in insulin-treated type 2 diabetes, where CGM use was associated with modest but significant reductions in HbA1c [3,5]. The magnitude of HbA1c reduction in our study appears greater than that reported in randomized settings, likely reflecting the combined effects of CGM introduction, insulin optimization, and structured patient education. The stabilization of CGM-derived metrics after the initial phase suggests that early therapeutic adaptation is followed by maintenance of glycaemic control rather than continued incremental improvement.

In addition to glycaemic improvement, the observed reductions in total cholesterol and triglycerides suggest a broader cardiometabolic benefit associated with CGM-supported care and treatment intensification. Although CGM does not exert a direct lipid-lowering effect, these changes likely reflect improved metabolic control and behavioral adaptations facilitated by real-time glucose feedback. Previous studies have shown that improved glycaemic control in type 2 diabetes is associated with reductions in triglycerides and, to a lesser extent, total cholesterol, likely mediated through decreased hepatic glucose output and improved insulin sensitivity [17]. Similar patterns have been observed in interventional studies of insulin intensification and CGM-supported management, where improvements in glycaemic parameters were accompanied by favorable changes in triglyceride-rich lipoproteins [3,5]. In contrast, body weight and BMI remained relatively stable over the 12-month follow-up, despite intensification of insulin therapy. This finding is clinically relevant, as insulin intensification is typically associated with weight gain, primarily due to reduced glycosuria and anabolic effects of insulin [18]. The absence of significant weight increase in our cohort may therefore suggest that CGM-supported care facilitated better self-management and dietary adjustments, potentially mitigating this expected adverse effect. Renal parameters remained stable throughout follow-up, with no evidence of clinically meaningful deterioration in eGFR or albuminuria. While the duration of follow-up limits conclusions regarding long-term renal outcomes, these findings indicate that CGM-supported care is not associated with short-term renal harm in this population.

A clinically important finding of this study is the absence of increased hypoglycaemia despite significant improvement in glycaemic control. Time below range remained consistently low throughout follow-up, indicating that improved HbA1c was not achieved at the expense of increased hypoglycaemic burden. This is particularly relevant in insulin-treated populations, where fear of hypoglycaemia often limits treatment intensification. In randomized trials, CGM has been associated with reducing hypoglycaemia in type 1 diabetes and selected insulin-treated populations, although evidence in type 2 diabetes remains less consistent [3,11,19]. Our findings extend this evidence by suggesting that, in a real-world setting, CGM-supported care may enable safer glycaemic optimization without increasing hypoglycaemia risk. However, given the absence of baseline CGM-derived hypoglycaemia metrics and a comparator group, these findings should be interpreted as demonstrating maintenance of low hypoglycaemia burden in intensive insulin treated subjects rather than a reduction.

One of the most notable findings of this study is the progressive reduction in diabetes-related distress over time. This aligns with previous evidence indicating that CGM use may be associated with reducing emotional burden by improving treatment confidence, reducing uncertainty regarding glucose fluctuations, and facilitating more proactive self-management [11,12]. The continuous feedback provided by CGM could be associated with cognitive load associated with diabetes management and enhance patients’ sense of control, thereby contributing to improved psychological outcomes. Given the established association between distress and adverse clinical outcomes, including poor glycaemic control and reduced adherence, these findings may have important long-term implications for disease management [1,2].

For glycaemic outcomes, mixed-effects models demonstrated a consistent trend toward HbA1c reduction over time, although this did not reach conventional statistical significance after adjustment. Importantly, CGM-derived metrics, particularly glucose management indicator and glycaemic variability, remained the strongest correlates of HbA1c, reinforcing their clinical relevance in assessing glycaemic control

Socioeconomic status emerged as an important determinant of both distress and quality of life. Patients with lower income reported higher distress and poorer QoL, consistent with previous research demonstrating independent associations between socioeconomic disadvantage and adverse diabetes outcomes [20]. These findings likely reflect differences in access to healthcare resources, health literacy, and the financial burden associated with chronic disease management. Importantly, these results suggest that addressing socioeconomic disparities may be critical for improving patient-centered outcomes in diabetes care.

Quality of life improved significantly during follow-up and was more strongly associated with diabetes-related distress and socioeconomic factors than with HbA1c. This finding is consistent with prior studies demonstrating that psychosocial variables, rather than glycaemic control alone, are the primary determinants of perceived well-being in individuals with diabetes [9,19,21]. The lack of independent association between HbA1c and QoL in our study further supports the concept that improvements in metabolic control do not automatically translate into improved patient experience, highlighting the importance of integrating patient-reported outcomes into routine diabetes care [10].

Several trials have evaluated CGM in T2DM, but relatively few have examined both clinical and psychosocial outcomes longitudinally. Beck et al. demonstrated glycemic benefits without significant QoL improvements, potentially due to shorter follow-up (6 months) and lack of distress assessment [3]. In contrast, Polonsky et al. showed improved confidence, reduced regimen distress, and fewer diabetes-related worries within 3 months of CGM use [11]. Our findings extend this body of evidence by providing longer-term real-world data over 12 months, demonstrating not only sustained improvements in glycaemic control but also consistent reductions in diabetes-related distress and meaningful enhancements in quality of life. These results support the concept that the psychosocial benefits of CGM may become more apparent over time and may require longer follow-up to be fully captured.

Continuous glucose monitoring (CGM) is increasingly recognized as extending beyond its traditional role as a glucose monitoring tool, contributing to a broader behavioral and digital therapeutic approach in diabetes care. Contemporary management strategies now incorporate advanced metrics such as time in range alongside HbA1c, reflecting a shift toward more nuanced and patient-centered assessment of glycaemic control [13,14]. Within this framework, CGM may influence not only metabolic outcomes but also patient behavior, treatment engagement, and psychosocial well-being.

This study adds to the existing literature by providing long-term real-world evidence on the combined metabolic and psychosocial impact of CGM-supported care in insulin-treated type 2 diabetes [22]. The integration of longitudinal clinical and patient-reported outcomes provides a more holistic understanding of treatment effects and highlights the importance of addressing both physiological and psychological dimensions of disease management.

## 5. Conclusions

In adults with type 2 diabetes treated with intensified insulin therapy, implementation of CGM as part of a structured care pathway was associated with sustained improvement in glycaemic control, reduced diabetes-related distress, and improved diabetes-related quality of life over 12 months. Given the observational design and the concurrent use of insulin optimization and patient education, these findings should be interpreted as real-world associations within a multimodal care model rather than as isolated causal effects of CGM. Further controlled and multicenter studies are warranted to confirm these observations and to better delineate the independent contribution of CGM to clinical and patient-reported outcomes.

### Limitations and Strengths

This study has several limitations that should be considered when interpreting the findings. First, because CGM was introduced as part of a broader therapeutic intensification strategy, including insulin dose optimization and structured education, the independent effect of CGM cannot be isolated. The findings should consequently be interpreted as associations observed within a CGM-supported structured care pathway rather than as causal effects of CGM alone. Second, the study was conducted at a single tertiary center, which may limit generalizability to other healthcare settings or populations with different demographic and socioeconomic characteristics, and patients who remained in follow-up for 12 months may have differed from those who missed visits or discontinued follow-up in motivation, health literacy or treatment engagement. Socioeconomic factors may have influenced both access to diabetes-related resources and patient-reported outcomes, particularly distress and quality of life. Finally, baseline CGM-derived metrics were not available, restricting the ability to directly assess changes in glycaemic variability and hypoglycaemia exposure from pre-intervention levels. These limitations are inherent to the real-world observational design but are also clinically informative, as they reflect the complexity of CGM implementation in routine care. From a methodological perspective, linear mixed-effects models were used to account for repeated measures with incomplete follow-up; however, these models assume missing at random, which cannot be formally verified in this real-world dataset.

Despite these limitations, several strengths enhance the validity and clinical relevance of the findings. The longitudinal design with repeated measurements over 12 months allows for assessment of both short- and longer-term effects of CGM-supported care. The integration of clinical and patient-reported outcomes provides a comprehensive evaluation of both metabolic and psychosocial dimensions of diabetes management. The use of validated instruments and standardized follow-up intervals further strengthens internal consistency, while the real-world setting increases the applicability of results to routine clinical practice.

## Figures and Tables

**Table 1:** Baseline Characteristics of Study Participants.

Baseline Characteristic	
Anthropometrics	Median (IQR)
BMI (kg/m^2^)	28.70 (26.6–31.8)
Body weight (kg)	83.65 (73.6–97.0)
Height (cm)	171.0 (163.0–177.5)
Diabetes-related	Median (IQR)
Duration of diabetes (months)	218 (120–288)
Initial Laboratory Parameters	Median (IQR)
HbA1c (%)	8.1 (7.3–9.5)
Fasting glucose (mmol/L)	8.4 (6.7–10.7)
Total cholesterol (mmol/L)	4.21 (3.59–5.18)
LDL cholesterol (mmol/L)	2.30 (1.62–3.00)
HDL cholesterol (mmol/L)	1.20 (0.94–1.40)
Triglycerides (mmol/L)	1.58 (1.10–2.48)
eGFR (mL/min/1.73 m^2^)	73.5 (61.8–96.0)
UACR (mg/g)	5.8 (1.0–34.8)
Lifestyle factors	*n* (%)
Smoking	
No	153 (67.7)
Yes	50 (22.2)
Former	23 (10.3)
Alcohol use	
None	191 (84.6)
Occasional	28 (12.2)
Regular	7 (3.2)
Comorbidities	*n* (%)
Hypertension	158 (70.1)
Hyperlipidemia	169 (74.7)
Complications	*n* (%)
Micro and macrovascular complications	134 (59.5)

Body mass index (BMI), Glycated hemoglobin (HbA1c), High-density lipoprotein (HDL), Low-density lipoprotein (LDL), Estimated glomerular filtration rate (eGFR), Urine albumin-to-creatinine ratio (UACR), Interquartile range (IQR).

**Table 2:** Longitudinal Changes in Metabolic and Laboratory Parameters During Follow-up.

Metabolic and Laboratory Parameters	Median (IQR)	Median (IQR)	* *p*	Median (IQR)	* *p*	Median (IQR)	* *p*	Median (IQR)	* *p*
	0 Month	3 Months	3 vs. 0	6 Months	6 vs. 0	9 Months	9 vs. 0	12 Months	12 vs. 0
Body weight (kg)	83.7 (73.6–97.0)	84.5 (73.0–95.0)	0.043	84.0 (72.0–95.0)	0.001	82.5 (70.0–92.8)	0.121	78.0 (68.0–90.0)	0.139
BMI (kg/m^2^)	28.7 (26.6–31.8)	28.9 (26.5–31.9)	0.106	29.0 (26.3–31.8)	0.012	29.0 (25.8–31.5)	0.140	27.1 (25.3–31.1)	0.574
Fasting glucose (mmol/L)	8.4 (6.7–10.7)	7.8 (6.4–9.6)	0.010	7.6 (6.5–9.2)	0.083	7.6 (6.6–9.5)	0.094	8.3 (6.0–9.7)	0.324
HbA1c (%)	8.1 (7.3–9.5)	7.2 (6.6–7.9)	<0.001	7.2 (6.7–7.9)	<0.001	7.1 (6.6–7.9)	<0.001	7.1 (6.5–7.7)	<0.001
Total cholesterol (mmol/L)	4.21 (3.59–5.18)	3.72 (3.07–4.40)	<0.001	3.65 (3.00–4.50)	<0.001	3.49 (2.87–4.29)	<0.001	3.44 (2.99–4.19)	<0.001
Triglycerides (mmol/L)	1.58 (1.10–2.48)	1.23 (0.91–1.87)	<0.001	1.29 (0.95–2.10)	0.003	1.31 (0.92–2.08)	<0.001	1.32 (0.88–1.78)	0.006
HDL (mmol/L)	1.20 (0.94–1.40)	1.22 (1.05–1.50)	0.072	1.25 (1.02–1.44)	0.160	1.19 (0.97–1.41)	0.683	1.19 (1.00–1.47)	0.473
LDL (mmol/L)	2.30 (1.62–3.00)	2.35 (1.81–2.89)	0.021	2.22 (1.78–2.94)	0.129	2.12 (1.71–2.77)	0.581	2.15 (1.80–2.72)	0.877
eGFR (mL/min/1.73 m^2^)	73.5 (61.8–96.0)	82.0 (62.0–96.8)	0.326	78.0 (62.5–96.3)	0.979	70.0 (58.0–95.0)	0.131	79.5 (64.3–95.8)	0.486
UACR (mg/g)	5.8 (1.0–34.8)	1.4 (0.7–14.0)	0.047	5.3 (3.1–18.1)	0.109	1.8 (0.9–2.6)	0.062	2.3 (0.9–5.1)	0.084

* *p* values represent pairwise comparisons between each follow-up visit and baseline (0 month). Post hoc pairwise comparisons between baseline and each follow-up visit were performed using the Wilcoxon signed-rank test; Body mass index (BMI), Glycated hemoglobin (HbA1c), High-density lipoprotein (HDL), Low-density lipoprotein (LDL), Estimated glomerular filtration rate (eGFR), Urine albumin-to-creatinine ratio (UACR), Interquartile range (IQR).

**Table 3:** Longitudinal Changes in CGM-Derived Glucose Metrics.

	Median (IQR)						
	3 month	6 month	9 month	12 month	* *p* (3 vs. 6)	* *p* (3 vs. 9)	* *p* (3 vs. 12)
GMI	7.1 (6.7–7.5)	7.1 (6.7–7.6)	7.1 (6.7–7.6)	7.0 (6.6–7.7)	0.575	0.747	0.354
TAR	23.0 (13.3–35.0)	23.0 (13.0–35.0)	24.0 (16.0–33.0)	21.5 (13.3–32.8)	0.658	0.897	0.006
TIR	73.5 (56.3–83.8)	71.0 (53.0–85.0)	72.0 (55.0–81.0)	73.5 (53.3–83.0)	0.911	0.745	0.109
TBR	0.0 (0.0–1.0)	0.0 (0.0–1.0)	0.0 (0.0–1.0)	0.0 (0.0–1.8)	0.872	0.323	0.017
CV	26.8 (23.7–30.9)	26.7 (23.7–30.3)	26.7 (23.1–30.7)	27.1 (22.1–31.2)	0.992	0.984	0.277

* *p* values represent pairwise comparisons between each follow-up visit and baseline (0 month). Post hoc pairwise comparisons between baseline and each follow-up visit were performed using the Wilcoxon signed-rank test; Glucose management indicator (GMI), Time above range (TAR), Time in range (TIR), Time below range (TBR), Coefficient of variation (CV), Interquartile range (IQR).

**Table 4:** Linear mixed-effects models of longitudinal outcomes.

Outcome	Variable	β	*p*-Value
HbA1c	Time	−0.016	0.076
	GMI	+0.602	<0.001
	TAR	+0.007	0.082
	CV	+0.014	0.020
Distress	Time	−0.025	0.001
	Age	+0.022	0.002
	Income	−0.666	0.027
QoL	Time	—	<0.05
	Distress	—	<0.05
	Income	—	<0.05

Linear mixed-effects models with random intercept for participant. Glycated hemoglobin (HbA1c), Glucose Management Indicator (GMI); Time Above Range (TAR); Coefficient of Variation (CV); Quality of Life (QoL).

## Data Availability

All data are available upon request.

## Abbreviations

The following abbreviations are used in this manuscript:

UACR	Urine albumine-creatinine ratio
T2DM	Type 2 diabetes mellitus
BMI	Body mass index
HbA1c	Glycated hemoglobin
eGFR	Estimated glomerular filtration rate
HDL	High-density lipoprotein
LDL	Low-density lipoprotein
